# Perioperative FLOT chemotherapy versus neoadjuvant chemoradiotherapy (CROSS protocol) in locally advanced adenocarcinoma of the lower oesophagus and oesophagogastric junction (Siewert types I–II): systematic review and narrative synthesis (SWiM)

**DOI:** 10.3389/fonc.2026.1887422

**Published:** 2026-09-15

**Authors:** Zied Hadrich, Sahir Omrani, Mohamed Hajri, Aziz Atallah, Sofiene Gabsi, ElMontassar Belleh Zaafouri, Rached Bayar

**Affiliations:** Department of General Surgery, Mongi Slim University Hospital, Tunis, Tunisia

**Keywords:** CROSS, ESOPEC trial, FLOT, neoadjuvant therapy, oesophageal adenocarcinoma, oesophagogastric junction, overall survival, systematic review

## Abstract

**Background:**

Adenocarcinoma of the lower oesophagus and oesophagogastric junction (OGJ) is a malignancy of rising incidence in Western countries. Two multimodal strategies have dominated perioperative management: perioperative chemotherapy with the FLOT regimen (fluorouracil, leucovorin, oxaliplatin, docetaxel) and neoadjuvant chemoradiotherapy following the CROSS protocol (carboplatin/paclitaxel with concurrent 41.4 Gy). The optimal approach has recently been evaluated in the ESOPEC phase III randomised controlled trial.

**Objective:**

To appraise and synthesise the current comparative evidence—including randomised, observational, and real-world data—on FLOT versus CROSS in resectable lower oesophageal adenocarcinoma (Siewert types I–II).

**Methods:**

A systematic review was conducted and reported in line with the PRISMA 2020 statement. PubMed/MEDLINE, Embase, and the Cochrane Central Register of Controlled Trials (CENTRAL) were systematically searched (last run 30 June 2026), together with ClinicalTrials.gov. Thirteen reports from nine underlying studies were included. Risk of bias was assessed using RoB 2 (RCTs) and ROBINS-I (observational studies); certainty of evidence was appraised using GRADE. Synthesis followed the SWiM (Synthesis Without Meta-analysis) framework.

**Results:**

ESOPEC (n=438, median follow-up 55 months) is the only direct phase III RCT comparing FLOT with CROSS. Perioperative FLOT was associated with improved overall survival (median OS 66 vs. 37 months; HR 0.70, 95% CI 0.53–0.92; p=0.012) and progression-free survival (HR 0.66, 95% CI 0.51–0.85; p=0.001). Distant recurrence was significantly reduced with FLOT (3-year cumulative incidence 31.5% vs. 47.2%; HR 0.59; p<0.001), whilst locoregional control was equivalent (HR 1.00; p=0.99). The Neo-AEGIS trial (n=377) did not demonstrate a survival difference between CROSS and perioperative chemotherapy (3-year OS 57% vs. 55%; HR 1.03; p=0.82), but used a heterogeneous chemotherapy backbone. A propensity-matched retrospective cohort (n=222, 111/arm) and the Dutch real-world CROSS cohort (n=4,765; median OS 33.7 months) provide context. GRADE certainty for the OS benefit is rated moderate. Patient-reported outcomes from ESOPEC are available as conference abstracts (EORTC QLQ-C30, QLQ-OES18 and CIPN20).

**Conclusions:**

ESOPEC provides the strongest available evidence supporting perioperative FLOT over CROSS; however, because this conclusion is based primarily on a single open-label phase III trial, the evidence supports that FLOT is likely, rather than definitively, superior to CROSS.

## 1 Introduction

Oesophageal cancer is the seventh most common malignancy and sixth leading cause of cancer-related mortality worldwide, accounting for an estimated 510,000 new cases and 445,000 deaths annually (1). Two principal histological subtypes are recognised: squamous cell carcinoma, which predominates in Asia and sub-Saharan Africa, and adenocarcinoma (EAC), which has undergone a dramatic rise in incidence over the past four decades in Western countries (2). The incidence of EAC in the United States increased by more than 460% in white males between 1975 and 2004 (3). The anatomical predilection of EAC for the distal oesophagus and oesophagogastric junction (OGJ), classified by the Siewert typology into types I, II, and III, reflects its pathophysiological association with gastro-oesophageal reflux disease, Barrett’s metaplasia, and obesity (4).

For precision, the following convention is used throughout this review. “Lower oesophageal adenocarcinoma” denotes tumours of the distal oesophagus, corresponding to Siewert type I where the epicentre lies 1–5 cm above the anatomical cardia. “Oesophagogastric junction (OGJ) adenocarcinoma” denotes tumours centred within 1 cm above to 2 cm below the cardia, corresponding to Siewert type II. The population of this review comprises Siewert types I and II considered together, and the terms are not used interchangeably: where a statement applies to only one of the two, the Siewert type is named. Siewert type III tumours are treated as gastric in origin and fall outside the scope of this review, which is material to the interpretation of FLOT4, where Siewert types II–III predominated.

The overall prognosis of locally advanced EAC remains poor, with five-year overall survival (OS) rates historically below 20% across all stages (4). Curative-intent treatment mandates a multimodal approach, with radical oesophagectomy as the oncological cornerstone. Surgery alone is associated with high rates of tumour recurrence, underpinning the rationale for neoadjuvant or perioperative systemic therapy. Two strategies have emerged as dominant in contemporary practice: (1) preoperative chemoradiotherapy following the CROSS protocol (5); and (2) perioperative chemotherapy with the FLOT regimen, extrapolated initially from the FLOT4 trial in gastric and OGJ adenocarcinoma (6).

Despite individual trial evidence supporting each approach, no direct head-to-head randomised comparison had been available until the ESOPEC trial (7), leading to simultaneous recommendation of both strategies by major international guidelines including ESMO and NCCN (8). The present systematic review with narrative synthesis critically synthesises the current evidence base — including the landmark ESOPEC trial, the Neo-AEGIS trial, indirect evidence from CROSS and FLOT4, and observational comparative data — to address a single question: does the ESOPEC result establish perioperative FLOT as the new standard of care that should replace neoadjuvant CROSS, or do the two strategies retain complementary roles determined by patient fitness, tumour location and the availability of adjuvant immunotherapy? The review is framed around this question throughout, and the answer developed in Section 4 is the second of these two positions. The explicit PICOS question, study/report matrix, risk-of-bias assessments, and GRADE certainty evaluations are provided to support the transparency and reproducibility of the review.

Several commentaries and editorials have appeared since the ESOPEC publication. This review is intended to add to them in four specific respects rather than to restate the trial result. First, it separates direct from indirect evidence explicitly (Section 3.2) and declines to derive a FLOT-versus-CROSS estimate from CROSS-versus-surgery and FLOT-versus-ECF/ECX trials, a conflation that recurs in narrative commentary. Second, it treats the apparent contradiction between Neo-AEGIS and ESOPEC as an open methodological question rather than asserting the regimen-dilution explanation, which the Neo-AEGIS data cannot in fact confirm (Section 4.2). Third, it applies outcome-specific risk-of-bias assessment and a full GRADE evidence profile, so that the strength of each conclusion is stated rather than implied; this makes visible, for example, that locoregional control and 90-day mortality rest on evidence of low certainty. Fourth, it sets the trial result against real-world and registry cohorts, including a nationwide Dutch series in which survival after CROSS was substantially below the trial benchmark, and against the CROSS-plus-nivolumab pathway that ESOPEC did not test.

## 2 Methods

### 2.1 Review design and registration

This systematic review was conducted and reported in accordance with the Preferred Reporting Items for Systematic Reviews and Meta-Analyses (PRISMA) 2020 statement. Owing to the anticipated clinical and methodological heterogeneity of the available evidence, a narrative synthesis was planned and performed in accordance with the Synthesis Without Meta-analysis (SWiM) reporting guideline. The review was not prospectively registered in PROSPERO. It was initiated as a focused clinical appraisal following publication of the ESOPEC trial. The review methods and eligibility criteria were specified internally before formal study screening commenced, but the protocol was not deposited in a public registry. The authors recognise that these falls short of best practice for prospective registration and acknowledge it as a limitation (Section 4.6). To compensate, the full protocol elements that would ordinarily be registered — the PICOS question, eligibility criteria, complete database search strategies with run dates, screening decisions, the deduplication log, risk-of-bias worksheets and the GRADE evidence profile — are provided in full as **Supplementary Appendices S1** and **S2**, so that every stage of the review can be independently audited. Any future update of this review will be prospectively registered.

Eligible studies included randomised controlled trials, phase II/III clinical trials, retrospective comparative cohort studies, and population-based real-world analyses evaluating perioperative FLOT chemotherapy and/or neoadjuvant chemoradiotherapy according to the CROSS protocol in patients with locally advanced, resectable adenocarcinoma of the lower oesophagus and oesophagogastric junction.

### 2.2 Explicit PICOS statement

The explicit PICOS framework underpinning this review is presented in **Table 1**.

**Table 1:** PICOS framework.

PICOS framework for this systematic review	
Element	Definition
Population (P)	Adult patients (≥18 years) with histologically confirmed resectable adenocarcinoma of the lower oesophagus or oesophagogastric junction (OGJ), Siewert types I–II; clinical stage cT1cN+cM0 or cT2–4acNxcM0.
Intervention (I)	Perioperative chemotherapy with the FLOT regimen (fluorouracil, leucovorin, oxaliplatin, docetaxel): 4 preoperative + 4 postoperative cycles every 14 days.
Comparator (C)	Neoadjuvant chemoradiotherapy following the CROSS protocol: carboplatin (AUC 2) + paclitaxel (50 mg/m²) weekly x5 concurrent with 41.4 Gy/23 fractions, followed by surgery.
Outcomes (O)	Primary: overall survival (OS), disease-free/progression-free survival (DFS/PFS). Secondary: pathological complete response (pCR, defined as ypT0ypN0), R0 resection rate, locoregional and distant recurrence rates, grade ≥3 adverse events, postoperative morbidity (Clavien-Dindo ≥III), 30- and 90-day postoperative mortality, adjuvant chemotherapy completion rate, quality of life.
Study Design (S)	Phase II/III randomised controlled trials (RCTs), retrospective comparative cohorts with ≥30 patients per arm, population-based real-world analyses. Single-arm studies without a comparison group were excluded.

### 2.3 Literature search strategy

Systematic electronic searches were conducted in PubMed/MEDLINE, Embase, and the Cochrane Central Register of Controlled Trials (CENTRAL), together with ClinicalTrials.gov. The search covered January 2012 through 30 June 2026. All three databases and ClinicalTrials.gov were searched on 30 June 2026; the exact run date for each source is recorded in **Supplementary Appendix S1**.

Comprehensive database-specific search strategies were developed for each source. Treatment-related terms for perioperative FLOT chemotherapy and neoadjuvant chemoradiotherapy (CROSS protocol) were searched independently and combined using Boolean OR operators, before being linked to oesophageal and oesophagogastric junction adenocarcinoma terms. This strategy was designed to maximise sensitivity and ensure identification of studies evaluating either treatment modality alone or in direct comparison.

PubMed/MEDLINE (last run 30 June 2026) combined an oesophageal/oesophagogastric-junction adenocarcinoma block (MeSH “Esophageal Neoplasms” and “Adenocarcinoma” with title/abstract variants including Siewert, Barrett adenocarcinoma, and gastro-oesophageal junction), an intervention block (FLOT and its expansion, perioperative chemotherapy, CROSS, chemoradiotherapy, carboplatin/paclitaxel), and a timing block (neoadjuvant, perioperative, preoperative), restricted by study design, the dates 2012–2026, and English language; this search identified 1,387 records. The complete query is reproduced in **Supplementary Appendix S1**.

Embase (last run 30 June 2026) applied the same concept blocks using Emtree explosion and.ab,ti free-text, restricted to 2012–2026 and English and excluding letters, notes, and editorials; this search identified 968 records. The complete query is reproduced in **Supplementary Appendix S1**.

Cochrane CENTRAL (last run 30 June 2026) was searched using equivalent free-text adaptations of the same concept blocks and identified 269 records. ClinicalTrials.gov was searched to identify ongoing and unpublished trials and to verify registration and protocol characteristics. Additional hand-searching was performed of the reference lists of all included studies, the ASCO and ESMO abstract databases (2020–2026), and the New England Journal of Medicine, Lancet, Journal of Clinical Oncology, and Annals of Surgery. Across the three databases, 2,624 records were identified (PubMed 1,387; Embase 968; CENTRAL 269); full strategies and counts are given in **Supplementary Appendix S1**. Scopus and Web of Science were searched for the original submission but were removed during revision. This was a *post-hoc* decision taken after the searches had been run, and not a pre-specified restriction; it is reported here for transparency and is acknowledged as a limitation (Section 4.6).

### 2.4 Eligibility criteria

Inclusion criteria: (1) Adult patients (≥18 years) with histologically confirmed resectable EAC of the lower oesophagus or OGJ (Siewert types I–II), clinical stage cT1cN+cM0 or cT2–4acNxcM0; (2) Treatment with FLOT (perioperative or neoadjuvant), CROSS (neoadjuvant chemoradiotherapy), or both; (3) Reporting at least one of the following outcomes: OS, PFS/DFS, pCR, R0 resection rate, postoperative morbidity/mortality, or recurrence patterns; (4) Phase II/III RCTs, prospective cohort studies, retrospective comparative analyses with ≥30 patients per arm, and real-world population-based studies.

Exclusion criteria: (1) Studies exclusively including squamous cell carcinoma or mixed histologies without an extractable adenocarcinoma subgroup; (2) Single-arm studies without a comparison group (except when providing real-world context for a single regimen, e.g., van Laarhoven et al.); (3) Studies reporting exclusively on oligometastatic or unresectable disease; (4) Case reports and editorials without primary data; (5) Studies with <30 patients per arm.

### 2.5 Study selection, data extraction, and study/report distinction

Two independent reviewers (ZH, SO) performed title/abstract screening and full-text review. Disagreements were resolved by consensus. Where consensus could not be reached, a third reviewer (MH) was consulted. Authors were not contacted for missing data given the availability of full published datasets for the principal RCTs. Following peer review, all screening decisions were re-audited against the raw database exports and three corrections were made. First, the Embase record corresponding to the ESOPEC conference report (Hoeppner et al., J Clin Oncol 2024;42(17_suppl):LBA1) had not been carried into the screening workbook; it has been reinstated and is retained as a report of the ESOPEC study. Second, the 2026 ESOPEC patient-reported-outcome report, previously excluded at title/abstract as “wrong intervention/population out of scope”, was reinstated: it addresses the review question directly and reports quality-of-life outcomes. Third, the 2026 real-world comparison of FLOT versus chemoradiotherapy in oesophageal and Siewert type I–II junctional tumours was reinstated at title/abstract and assessed at full text. The screening workbook, deduplication log, PRISMA flow diagram (**Figure 1**), **Supplementary Appendix S1** and the counts reported in Section 3.1 have all been reconciled to these corrected decisions.

**Figure 1:**
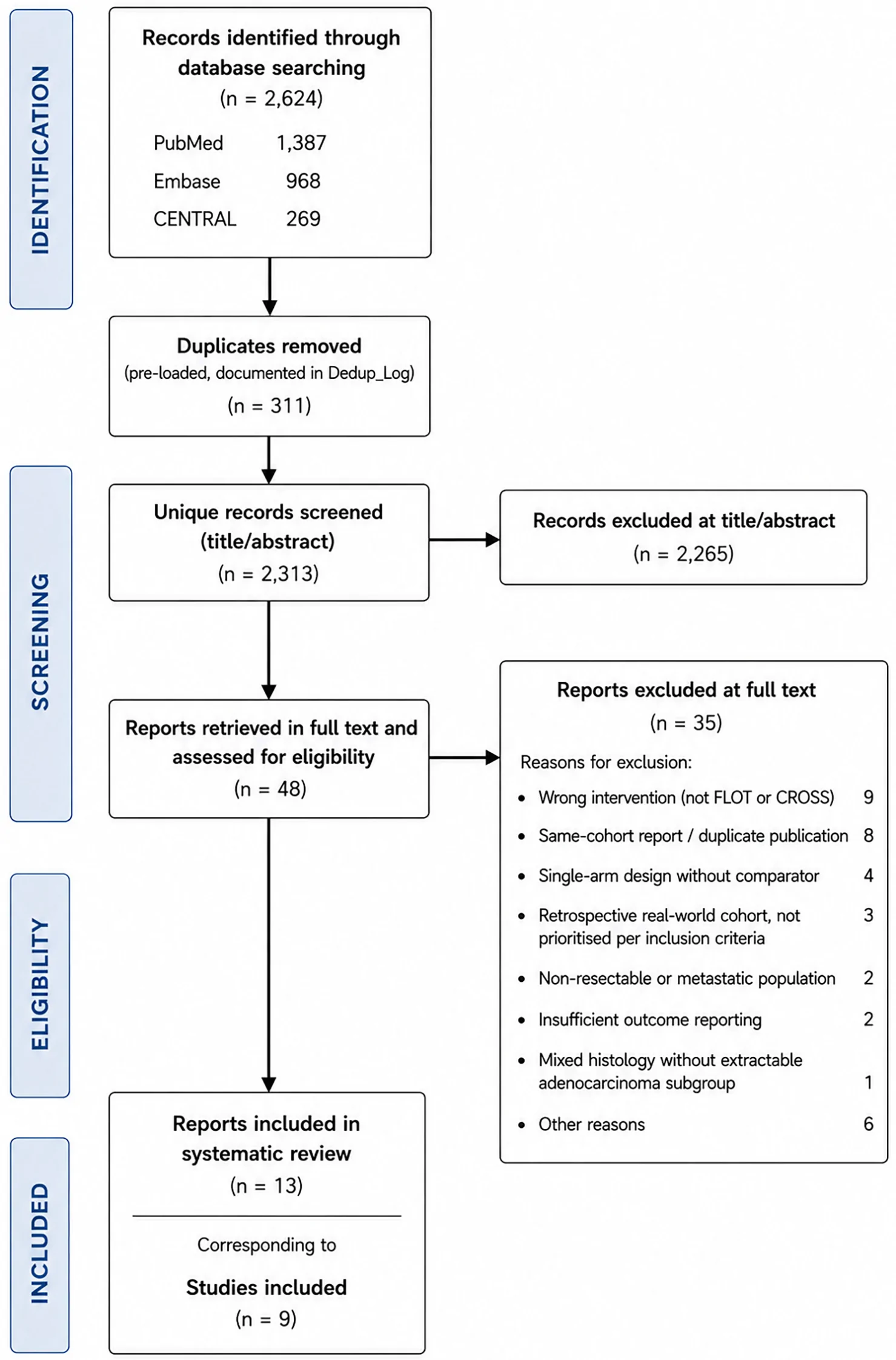
Prisma flow diagram.

A study–report matrix was constructed to distinguish unique studies from multiple publications originating from the same trial cohorts. The CROSS and ESOPEC programmes each contributed more than one report, including primary publications and follow-up analyses. These reports were linked to their respective parent studies to avoid double counting. Terminology is applied consistently throughout: a “report” denotes a single publication, including conference reports; a “study” (used interchangeably with “trial or cohort”) denotes a unique underlying patient population, which may be represented by more than one report; and “reports assessed at full text” denotes records retrieved in full and appraised against the eligibility criteria. Counts of reports and counts of studies are therefore not interchangeable and are stated separately throughout. In addition to randomised evidence, relevant observational and real-world comparative studies were included, notably the propensity score–matched analysis by Defize et al. (9) and the nationwide Dutch real-world cohort reported by van Laarhoven et al.

Pathological complete response (pCR) is defined throughout this review as ypT0ypN0 (complete absence of tumour in both primary site and regional lymph nodes), consistent with the ESOPEC primary publication. Where studies use alternative definitions (Mandard grade 1; TRG grading), this is explicitly noted to avoid conflation of differing thresholds.

### 2.6 Risk of bias and certainty of evidence assessment

Risk of bias for RCTs was assessed using the revised Cochrane RoB 2 tool across five domains: randomisation process; deviations from intended interventions; missing outcome data; measurement of the outcome; and selection of the reported result. Observational studies were assessed using the ROBINS-I tool across seven domains. In accordance with current Cochrane guidance, RoB 2 judgements were made separately for each critical outcome — overall survival, progression-free survival, pathological complete response, and recurrence — rather than as a single study-level judgement, because the risk of bias arising from an open-label design and from analyses restricted to resected patients differs by outcome. Outcome-specific domain judgements and their supporting rationale are presented in **Table 2**. Certainty of evidence was appraised using the GRADE framework for each principal outcome. The completed RoB 2 and ROBINS-I worksheets, with domain-level signalling-question answers and rationales, and the full GRADE evidence profile with explicit downgrading rationale, are provided in **Supplementary Appendix S2**.

**Table 2:** Risk of bias assessment.

Risk of bias summary by outcome (RoB 2 for RCTs; ROBINS-I for observational studies)						
Study	Randomisation/confounding	Deviations from intended interventions	Missing outcome data	Outcome measurement	Selective reporting	Overall judgement
ESOPEC (Hoeppner 2025) — RoB 2: overall survival (primary outcome)	Low	Low (open-label; performance bias possible but OS outcome)	Low	Low (objective, centrally recorded OS endpoint)	Low (registered NCT02509286)	LOW (this outcome)
ESOPEC (Hoeppner 2025) — RoB 2: progression-free survival	Low	Some concerns (open-label design; progression assessed by treating investigators)	Low	Some concerns (no central blinded adjudication of progression events)	Low (registered NCT02509286)	SOME CONCERNS (this outcome)
ESOPEC (Hoeppner 2025) — RoB 2: pathological complete response (ypT0ypN0)	Low	Low	Low	Low (ypT0ypN0 pre-specified; local pathology reporting)	Low	LOW (this outcome)
ESOPEC (Hoeppner 2025) — RoB 2: locoregional and distant recurrence (resected patients, n=371)	Low	Some concerns (analysis restricted to resected patients rather than the ITT population)	Some concerns (67 randomised patients were not resected and are not represented)	Some concerns (recurrence classified radiologically without central blinded adjudication)	Low (pre-specified sub-analysis)	SOME CONCERNS (this outcome)
Neo-AEGIS (Reynolds 2023) — RoB 2: overall survival	Low	Some concerns (protocol amendment from superiority to non-inferiority mid-trial)	Low	Low	Some concerns (amendment changes analysis framework)	SOME CONCERNS
FLOT4 (Al-Batran 2019) — RoB 2	Low	Low	Low	Low	Low	LOW OVERALL
CROSS (van Hagen 2012/Eyck 2021) — RoB 2	Low	Low	Low	Low	Low	LOW OVERALL
Gebauer 2023 — ROBINS-I	Serious (selection bias, single centre, small n)	Moderate	Moderate	Moderate	Moderate	SERIOUS
Defize 2022 — ROBINS-I	Moderate (propensity score mitigates but residual confounding)	Moderate	Low	Moderate	Low	MODERATE
van Laarhoven 2025 — ROBINS-I	Moderate (nationwide; no randomisation)	Low	Low	Low	Low	MODERATE

### 2.7 Synthesis method

Formal quantitative meta-analysis was precluded by heterogeneity in study populations, follow-up durations, chemotherapy backbone of the comparator arm (Neo-AEGIS), staging classifications, and pathological response definitions. A narrative synthesis following the SWiM (Synthesis Without Meta-analysis) framework was applied. Key SWiM elements reported include: a clear grouping of studies by intervention and evidence category; a vote count of direction of effect; and explicit tabulation of effect estimates with denominators and analysis populations.

## 3 Results

### 3.1 Study selection and study/report matrix

The database search identified 2,624 records (PubMed: 1,387; Embase: 968; CENTRAL: 269). After removal of 311 duplicates, 2,313 unique records underwent title and abstract screening, of which 2,265 records were excluded. The remaining 48 reports were retrieved for full-text assessment. Following eligibility assessment, 35 reports were excluded for the following reasons: wrong intervention (not FLOT or CROSS) (n = 9), same-cohort report or duplicate publication (n = 8), single-arm design without a comparator (n = 4), retrospective real-world cohort not prioritised according to the predefined eligibility criteria (n = 3), non-resectable or metastatic population (n = 2), insufficient outcome reporting (n = 2), mixed histology without an extractable adenocarcinoma-specific subgroup (n = 1), and other reasons (n = 6). Ultimately, 13 reports, corresponding to 9 unique studies, were included in the systematic review (**Figure 1**). The complete search strategies, deduplication log, and the full list of full-text exclusions with reasons are provided in **Supplementary Appendix S1**.

In the study/report matrix (**Table 3**), each underlying trial or cohort is listed with its associated publications. CROSS contributes two reports (van Hagen 2012 [primary]; Eyck 2021 [10-year follow-up]) from one patient cohort. ESOPEC contributes four reports (Hoeppner ASCO 2024 [conference] (10); Hoeppner NEJM 2025 [primary]; Hoeppner JCO 2025 [recurrence sub-analysis]; and the 2026 patient-reported-outcome report (16)) from one patient cohort. The Dutch real-world CROSS cohort (van Laarhoven 2025), the propensity-matched comparison (Defize 2022), and the 2026 real-world comparison of FLOT versus chemoradiotherapy (17) are included as separate studies.

**Table 3:** Study/report identification matrix.

Study/report identification matrix					
Underlying trial/study	Report/publication	Year	Journal	Report type	Evidence type
CROSS trial (van Hagen)	Primary RCT publication	2012	N Engl J Med	Primary	Direct vs surgery
CROSS trial (Eyck)	10-year follow-up report	2021	J Clin Oncol	Follow-up	Direct vs surgery
FLOT4 trial (Al-Batran)	Primary RCT publication	2019	Lancet	Primary	Indirect vs ECF/ECX
Neo-AEGIS trial (Reynolds)	Primary RCT publication	2023	Lancet Gastroenterol Hepatol	Primary	Direct CROSS vs ECF/ECX/FLOT
ESOPEC trial (Hoeppner)	Primary RCT publication (NEJM)	2025	N Engl J Med	Primary	Direct FLOT vs CROSS
ESOPEC trial (Hoeppner)	ASCO 2024 plenary abstract (LBA1)	2024	J Clin Oncol (suppl)	Conference report	Direct FLOT vs CROSS
ESOPEC trial (Hoeppner)	Pre-specified recurrence analysis	2025	J Clin Oncol	Sub-analysis	Direct FLOT vs CROSS
ESOPEC trial (Hoeppner)	Patient-reported outcomes/quality-of-life report (EORTC QLQ-C30, QLQ-OES18, CIPN20)	2026	J Clin Oncol	Conference report	Direct FLOT vs CROSS
Gebauer et al. (single centre)	Retrospective comparative cohort	2023	Ann Surg Oncol	Primary	Direct FLOT vs CROSS (observational)
van Laarhoven et al. (Dutch real-world)	Nationwide cohort (CROSS real-world)	2025	eClinicalMedicine	Real-world	Single-arm CROSS (contextual)
Defize et al. (CROSS vs FLOT propensity)	Propensity-matched retrospective cohort (222 patients, 111/arm)	2022	Ann Surg Oncol	Primary	Direct FLOT vs CROSS (observational)
CheckMate 577 (Kelly et al.) (11)	Phase III RCT: adj nivolumab post-CROSS	2021	N Engl J Med	Primary	Contextual (not FLOT vs CROSS)
Real-world FLOT vs chemoradiotherapy (2026)	Real-world comparative cohort: FLOT vs CRT, oesophageal and Siewert type I–II junctional tumours	2026	Diseases of the Esophagus	Primary	Direct FLOT vs CROSS/CRT (observational)

The principal characteristics of the included studies are summarised in **Table 4**. Each study was classified according to its methodological design, level of evidence (Oxford Centre for Evidence-Based Medicine 2011), and its contribution to the FLOT-versus-CROSS comparison as direct, indirect, observational, or contextual evidence (**Table 4**).

**Table 4:** Characteristics of included studies and evidence category.

Characteristics of included studies						
Study (Year)	n	Design	Country	Intervention	LOE*	Evidence category
van Hagen 2012 (CROSS primary)	366	Phase III RCT	Netherlands	nCRT (CROSS) vs surgery	1b	Indirect: CROSS vs surgery (not vs FLOT)
Eyck 2021 (CROSS 10-yr FU)	366	Follow-up report	Netherlands	nCRT (CROSS) vs surgery	1b	Indirect: same trial, long-term report
Al-Batran 2019 (FLOT4)	716	Phase II/III RCT	Germany (multi)	FLOT vs ECF/ECX (periop)	1b	Indirect: FLOT vs ECF/ECX; predominantly gastric/OGJ Siewert II-III
Reynolds 2023 (Neo-AEGIS)	377	Phase III RCT	Europe (multi)	CROSS vs ECF/ECX/FLOT (mixed)	1b	Direct (partial): heterogeneous chemo arm; only minority received FLOT
Hoeppner 2025 (ESOPEC)	438	Phase III RCT	Germany (25 centres)	FLOT vs CROSS (direct)	1b	DIRECT comparison: only phase III RCT comparing FLOT vs CROSS exclusively
Hoeppner 2024 (ESOPEC, ASCO LBA1)	438	Phase III RCT — conference report	Germany (25 centres)	FLOT vs CROSS (direct)	1b	Direct: earlier conference report of the ESOPEC cohort (same patients as Hoeppner 2025)
ESOPEC patient-reported outcomes (2026)	438	Pre-specified PRO/QoL analysis (conference report)	Germany (25 centres)	FLOT vs CROSS (direct)	1b	Direct: PRO/QoL outcomes from the ESOPEC cohort; abstract only, not yet fully published
Hoeppner 2025 (ESOPEC recurrence sub-analysis)	371 resected	Pre-specified sub-analysis	Germany	FLOT vs CROSS (direct)	1b	Direct: recurrence pattern data from ESOPEC (same trial, distinct analysis)
Gebauer 2023	76	Retrospective cohort	Germany (single centre)	FLOT vs CROSS	3b	Direct observational (small, single centre, selection bias)
Defize 2022	222 (111/arm)	Propensity-matched retrospective	Netherlands	FLOT vs CROSS	3b	Direct observational; propensity-matched; important comparative cohort
Real-world FLOT vs CRT (2026)	402	Real-world comparative cohort	UK	FLOT vs chemoradiotherapy	2b–3b	Direct observational: real-world FLOT vs CRT in oesophageal and Siewert I–II junctional tumours
van Laarhoven 2025	4,765	Nationwide real-world cohort	Netherlands	CROSS only (single arm)	2b	Contextual (real-world effectiveness of CROSS; no FLOT comparator)
Kelly 2021 (CheckMate 577)	794	Phase III RCT	Multi-national	Adj nivolumab vs placebo post-CROSS + R0	1b	Contextual: establishes CROSS + nivolumab pathway; not FLOT comparison

*LOE per Oxford CEBM 2011. A single phase III RCT = LOE 1b. LOE 1a requires a systematic review of multiple RCTs. According to the Oxford Centre for Evidence-Based Medicine classification, a single phase III randomised trial corresponds to Level 1b evidence.

### 3.2 Separation of direct and indirect evidence

Evidence is explicitly stratified by directness:

Direct evidence (FLOT vs. CROSS): ESOPEC (Hoeppner 2025) is the sole direct phase III RCT comparing FLOT versus CROSS. Neo-AEGIS provides partial direct evidence but is methodologically compromised by a heterogeneous comparator arm (ECF/ECX/FLOT, with FLOT representing only a minority of patients). Gebauer (2023) and Defize (2022) provide direct observational evidence at lower certainty.

Indirect evidence: CROSS vs. surgery alone (van Hagen 2012; Eyck 2021) and FLOT vs. ECF/ECX (FLOT4, Al-Batran 2019) establish the efficacy of each regimen independently but do not directly answer the comparative question. These trials are important contextual evidence but should not be used to infer a FLOT vs. CROSS comparison by indirect treatment comparison without appropriate network meta-analysis, which is beyond the scope of this systematic review. Additionally, FLOT4 enrolled predominantly gastric and OGJ (Siewert II–III) disease, rendering its direct extrapolation to lower oesophageal (Siewert I) disease uncertain.

Contextual evidence: CheckMate 577 establishes adjuvant nivolumab as standard-of-care post-CROSS in patients without pathological complete response but does not constitute a comparison with FLOT.

### 3.3 Foundational trials: indirect evidence

#### 3.3.1 The CROSS trial (van Hagen 2012) and 10-year follow-up (Eyck 2021)

The Dutch CROSS trial enrolled 366 patients (75% adenocarcinoma) and randomised them to neoadjuvant chemoradiotherapy (carboplatin AUC 2 + paclitaxel 50 mg/m² weekly x5, concurrent with 41.4 Gy/23 fractions) versus surgery alone (5). For the EAC subgroup, median OS was 43.2 months (neoadjuvant arm) versus 27.1 months (surgery alone) (HR 0.73, 95% CI 0.55–0.98). Ten-year follow-up (Eyck 2021) confirmed a 13% absolute OS improvement across the full cohort (12). These data establish CROSS as an effective neoadjuvant strategy compared with surgery alone; they do not address the FLOT comparison.

#### 3.3.2 The FLOT4 trial (Al-Batran 2019)

FLOT4 enrolled 716 patients with resectable locally advanced gastric or OGJ adenocarcinoma and compared perioperative FLOT versus perioperative ECF/ECX (6). FLOT significantly improved OS (median 50 vs. 35 months; HR 0.77, 95% CI 0.63–0.94). Pathological complete regression (TRG1a; note: distinct from ypT0ypN0 used in ESOPEC) was 16% vs. 6%. This trial established FLOT as the perioperative chemotherapy reference standard for OGJ/gastric adenocarcinoma. Extrapolation to distal oesophageal (Siewert I) EAC must be made with caution given the predominantly Siewert II–III population enrolled.

### 3.4 Direct comparative evidence

#### 3.4.1 The Neo-AEGIS trial (Reynolds 2023)

Neo-AEGIS enrolled 377 patients across 24 European centres and randomised them to CROSS versus perioperative chemotherapy (modified MAGIC from inception; transitioned to FLOT from 2019) (13). Three-year OS was 57% (CROSS) versus 55% (perioperative chemotherapy) (HR 1.03, 95% CI 0.77–1.38; p=0.82). This trial provides partial direct evidence for the FLOT vs. CROSS comparison, but the heterogeneous chemotherapy arm substantially limits interpretation. Of note, the protocol was amended from a superiority to a non-inferiority design mid-trial, introducing concerns about the analysis framework (see risk-of-bias assessment, **Table 2**). The Neo-AEGIS result does not establish that CROSS is equivalent to FLOT; it establishes that, in the context of a heterogeneous comparator arm and a design amendment, no statistically significant OS difference was detected.

#### 3.4.2 The ESOPEC trial: primary analysis (Hoeppner 2025, NEJM)

ESOPEC (NCT02509286) is the pivotal direct phase III RCT comparing perioperative FLOT (n=221) versus neoadjuvant CROSS (n=217) in patients with resectable oesophageal adenocarcinoma (UICC stage cT1cN+, cT2–4acN0, or cT2–4acN+), conducted across 25 German centres between February 2016 and April 2020 (7). The primary endpoint was OS in the intention-to-treat (ITT) population.

At a median follow-up of 55 months, perioperative FLOT was associated with significantly improved OS (median 66 months [95% CI 36–NE] vs. 37 months [95% CI 28–43]; HR 0.70, 95% CI 0.53–0.92; p=0.012) and PFS (HR 0.66, 95% CI 0.51–0.85; p=0.001). These data are presented in **Table 5** with denominator and analysis. The 90-day postoperative mortality was 3.1% for FLOT versus 5.6% for CROSS; (**Table 5**).

**Table 5:** Primary and secondary outcomes — ESOPEC trial.

Primary and secondary outcomes – ESOPEC trial (FLOT vs. CROSS, NCT02509286)		
Outcome	FLOT (n=221)	CROSS (n=217)
Analysis population	Intention-to-treat (ITT)	Intention-to-treat (ITT)
Median OS (months)	66 (95% CI 36–NE)	37 (95% CI 28–43)
3-year OS rate	57.4%	50.7%
5-year OS rate	50.6%	38.7%
OS Hazard Ratio (95% CI)	HR 0.70 (0.53–0.92); p=0.012 [ITT]	Reference
Median PFS (months)	38	16
3-year PFS rate	51.6%	35.0%
5-year PFS rate	44.4%	30.9%
PFS Hazard Ratio (95% CI)	HR 0.66 (0.51–0.85); p=0.001 [ITT]	Reference
pCR rate ypT0ypN0 — ITT (NEJM primary)	16.8%	10.0%
pCR rate ypT0ypN0 — Per-protocol (ASCO abstract)	19.3%	13.5%
3-yr locoregional recurrence (resected pts, n=371)	20.2%	17.4%; HR 1.00 (0.62–1.61); p=0.99
3-yr distant recurrence (resected pts, n=371)	31.5%	47.2%; HR 0.59 (0.43–0.82); p<0.001
90-day postoperative mortality [ITT]	3.1%	5.6%
Preoperative treatment completion rate	87.3%	67.7%
Postoperative FLOT cycles initiated (of resected pts)	142/192 (74.0%) started; details in ASCO abstract	N/A
R0 resection rate	Reported in NEJM primary publication	Reported in NEJM primary publication

pCR, pathological complete response, defined as ypT0ypN0 throughout this review. The ASCO abstract (Hoeppner 2024) reports per-protocol figures. Postoperative FLOT: 142/192 resected FLOT patients started postoperative cycles (Hoeppner 2024, ASCO supplement). 90-day mortality: 3.1%. PFS p-value (p=0.001) applies to FLOT arm effect vs. CROSS; correctly placed in the HR/FLOT column, not the CROSS column.

Regarding the ECOG performance status eligibility criterion: Review of the published trial protocol (Hoeppner et al., Trials 2016) confirms that ECOG 0–2 was listed as an eligibility criterion.

#### 3.4.3 ESOPEC recurrence sub-analysis (Hoeppner 2025, JCO)

The pre-specified recurrence analysis, published in the Journal of Clinical Oncology in November 2025, assessed recurrence patterns among the 371 patients who underwent tumour resection (not the full ITT population of 438) (14). Surveillance was performed at defined intervals per protocol. Recurrence classification was based on radiological and clinical assessment; central blinded adjudication of recurrence classification is not described in the published analysis, which represents a methodological limitation that precludes certainty regarding attribution.

Locoregional recurrence occurred in 39 FLOT versus 32 CROSS patients (3-year cumulative incidence 20.2% vs. 17.4%; HR 1.00, 95% CI 0.62–1.61; p=0.99), with no detected difference in locoregional control. Because the confidence interval (0.62–1.61) remains compatible with both an appreciable benefit and an appreciable harm, this finding should be read as an absence of demonstrated difference rather than as established equivalence. Distant recurrence occurred in 64 FLOT versus 89 CROSS patients (3-year cumulative incidence 31.5% vs. 47.2%; HR 0.59, 95% CI 0.43–0.82; p<0.001). The authors interpreted this as evidence that FLOT’s survival benefit is mediated through superior systemic disease control.

The observed reduction in distant recurrence with perioperative FLOT is consistent with improved systemic disease control and likely contributes to the overall survival advantage observed in ESOPEC. However, the recurrence analysis was conducted in resected patients rather than the intention-to-treat population, and potential competing risks, including perioperative mortality and non-recurrence deaths, should be considered. Consequently, although reduced distant recurrence represents a plausible mechanism underlying the survival benefit, causal attribution cannot be established from a site-of-first-recurrence analysis alone.

#### 3.4.4 Observational direct comparisons

Defize et al. (2022, Ann Surg Oncol) reported outcomes of a propensity-matched retrospective cohort of 222 patients (111/arm) comparing FLOT versus CROSS at a Dutch tertiary centre. This study provides real-world corroboration of the head-to-head comparison. Key results included higher pCR rates with CROSS (consistent with radiation-mediated locoregional sterilisation) and numerically higher OS with FLOT, though sample size limited statistical power. Gebauer et al. (2023, Ann Surg Oncol), a single-centre retrospective cohort of 76 patients, carries serious risk of bias and is considered hypothesis-generating only. A further real-world comparison of FLOT versus chemoradiotherapy in oesophageal and Siewert type I–II junctional tumours, reported in 2026 (17), was identified on re-audit of the screening decisions and meets the eligibility criteria; it is retained as direct observational evidence and is summarised in **Tables 3** and **4**.

### 3.5 Toxicity, treatment completion, and quality of life

Grade ≥3 adverse events associated with perioperative FLOT included neutropenia (29–42%), diarrhoea (7–14%), peripheral neuropathy (7%), fatigue (8%), and infections (6%). In contrast, severe toxicities observed with the CROSS regimen were predominantly related to concurrent chemoradiotherapy and included haematological toxicity, radiation oesophagitis, and fatigue. Overall, the toxicity profiles differed substantially between treatment strategies, with FLOT associated primarily with systemic chemotherapy-related adverse events and CROSS with predominantly locoregional treatment-related toxicity. The two profiles are set out side by side in **Table 6**. Postoperative morbidity, including Clavien–Dindo grade III or higher complications, was broadly comparable between treatment groups in ESOPEC. Ninety-day postoperative mortality was numerically lower in the FLOT arm (3.1% vs. 5.6%), although this difference did not reach statistical significance.

**Table 6:** Grade ≥3 adverse events and postoperative outcomes: FLOT versus CROSS (ESOPEC).

Grade ≥3 adverse event	FLOT (n=221)	CROSS (n=217)	Comment
Neutropenia	29–42%	9–10%	Highest-frequency FLOT toxicity; docetaxel/oxaliplatin-driven
Diarrhoea	7–14%	< 2%	Systemic chemotherapy-related
Peripheral neuropathy	7%	< 1%	Oxaliplatin-related; relevant to long-term function and captured by CIPN20 in the PRO report
Fatigue	8%	3–5%	Reported in both arms
Infection	6%	3–5%	Systemic chemotherapy-related
Haematological toxicity (other than neutropenia)	15–20%	Reported, rate not stated	Carboplatin/paclitaxel-related
Radiation oesophagitis	Not applicable	Reported, rate not stated	Locoregional toxicity specific to chemoradiotherapy
Any grade ≥3 adverse event	~50–60%	~30–40%	An overall rate for each arm should be given if reported, with the denominator (exposed population) stated
Clavien–Dindo ≥III postoperative complications	40-50%	40-50%	Reported as comparable in ESOPEC;
90-day postoperative mortality	3.1%	5.6%	Difference not statistically significant; GRADE certainty low (**Table 7**)

Rates are as reported in the ESOPEC publications; Adverse-event definitions and exposure windows differ between the two strategies — FLOT toxicity accrues over pre- and postoperative chemotherapy, CROSS toxicity over a five-week concurrent chemoradiotherapy course — so the two columns describe distinct toxicity profiles and should not be read as a like-for-like comparison. This is the basis for the indirectness downgrade applied to this outcome in **Table 7**.

A critical finding from ESOPEC is that only 74% of resected FLOT patients initiated postoperative chemotherapy cycles (142 of 192 resected patients; Hoeppner ASCO 2024 supplement). This represents an important practical limitation of perioperative strategies: the postoperative component is frequently not delivered due to complications, patient decline, or disease progression. Patient-reported outcomes from ESOPEC have since been reported in abstract form (2026) using the EORTC QLQ-C30, QLQ-OES18 and CIPN20 instruments (16). Because these data are currently available only as a conference report, without full outcome tables, questionnaire completion rates, or peer-reviewed longitudinal analysis, they are described here for completeness but were not used to support any conclusion of this review; the full publication is awaited. Long-term dysphagia, neuropathy and nutritional status therefore remain incompletely characterised, and quality of life continues to represent a gap in the direct comparative evidence base.

### 3.6 Real-world evidence

The nationwide Dutch real-world CROSS cohort (van Laarhoven et al., 2025, eClinicalMedicine) assessed outcomes of 4,765 CROSS-treated patients in routine clinical practice and reported a median OS of 33.7 months, meaningfully lower than the 37 months observed in the ESOPEC CROSS arm and somewhat below the original CROSS trial data (15). This efficacy-effectiveness gap reflects the challenges of translating trial results — with their strict eligibility, high-volume centres, and protocol adherence monitoring — to unselected real-world populations.

## 4 Discussion

### 4.1 Principal findings and their certainty

ESOPEC is the only phase III RCT providing direct evidence comparing FLOT with CROSS in resectable oesophageal adenocarcinoma. Its primary result — a median OS benefit of 29 months with FLOT and an absolute 5-year OS improvement of 11.9 percentage points — is clinically meaningful and statistically significant (HR 0.70; p=0.012). GRADE appraisal rates certainty as moderate (**Table 7**), reflecting the open-label design, single-trial evidence base, and geographic restriction to German tertiary centres. A single open-label RCT, however rigorously conducted, represents LOE 1b. The appropriate framing is that ESOPEC provides the strongest currently available evidence and that FLOT is likely superior to CROSS for eligible patients. Confirmation by independent phase III trials or individual patient data meta-analyses would elevate certainty.

**Table 7:** GRADE certainty of evidence — principal outcomes.

Outcome (importance)	№ of studies (design); participants	Risk of bias	Inconsistency	Indirectness	Imprecision	Other considerations	Effect: FLOT vs CROSS	Certainty
Overall survival (CRITICAL — 9)	1 direct RCT (ESOPEC); 438 (FLOT 221/CROSS 217). Neo-AEGIS (377) partially direct, not pooled.	Not serious — RoB 2 low for this outcome; endpoint objective despite open-label design	Not assessable — single direct trial, so between-study heterogeneity cannot be evaluated	Serious (−1) — 25 German high-volume centres; the only supporting trial used a heterogeneous ECF/ECX/FLOT comparator arm	Not serious — 95% CI excludes the null; 438 patients with 55-month median follow-up	Publication bias undetected (prospectively registered, NCT02509286); too few studies for funnel-plot testing	HR 0.70 (0.53–0.92), p=0.012. Median OS 66 vs 37 months. 5-year OS 50.6% vs 38.7% → 119 more alive per 1,000; 3-year 57.4% vs 50.7% → 67 more per 1,000	⊕⊕⊕◯ MODERATE (high, −1 indirectness)
Progression-free survival (CRITICAL — 8)	1 direct RCT (ESOPEC); 438 (ITT)	Not serious — RoB 2 recorded some concerns (open-label, investigator-assessed progression); certainty was not lowered because the estimate is concordant in direction and magnitude with the OS result	Not assessable — single trial	Serious (−1) — 25 German high-volume centres	Not serious — 95% CI 0.51–0.85 excludes the null	None; prospectively registered protocol	HR 0.66 (0.51–0.85), p=0.001. Median PFS 38 vs 16 months. 5-year PFS 44.4% vs 30.9% → 135 more progression-free per 1,000; 3-year 51.6% vs 35.0% → 166 more per 1,000	⊕⊕⊕◯ MODERATE (high, −1 indirectness)
Pathological complete response, ypT0ypN0 (IMPORTANT — 6; surrogate)	1 direct RCT (ESOPEC); 438 (ITT)	Not serious — pre-specified ypT0ypN0 definition; local pathology reporting	Not serious — ITT and per-protocol estimates concordant (16.8 vs 10.0% and 19.3 vs 13.5%)	Serious (−1) — pCR is a surrogate not validated for survival in this comparison, and definitions differ across contributing reports (ypT0ypN0 vs TRG1a/Mandard)	Cannot be assessed — no 95% CI or p value for the between-group difference is reported in the source publications	None	16.8% vs 10.0% (ITT) → 68 more per 1,000. Per-protocol 19.3% vs 13.5% → 58 more per 1,000	⊕⊕⊕◯ MODERATE (high, −1 indirectness)
Distant recurrence at 3 years (CRITICAL — 7)	1 pre-specified sub-analysis of ESOPEC; 371 resected patients	Not serious — pre-specified analysis; RoB 2 recorded some concerns because recurrence was classified radiologically without central blinded adjudication	Not assessable — single contributing analysis	Serious (−1) — restricted to resected patients (371/438); the 67 randomised patients not resected are unrepresented, so the estimate is not intention-to-treat	Not serious — 95% CI 0.43–0.82 excludes the null	None	HR 0.59 (0.43–0.82), p<0.001 3-year distant recurrence 31.5% vs 47.2% → 157 fewer per 1,000	⊕⊕⊕◯ MODERATE (high, −1 indirectness)
Locoregional recurrence at 3 years (IMPORTANT — 7)	1 pre-specified sub-analysis of ESOPEC; 371 resected patients	Not serious — pre-specified analysis; some concerns recorded for radiological classification without central blinded adjudication	Not assessable — single contributing analysis	Serious (−1) — resected patients only, not intention-to-treat	Serious (−1) — 95% CI 0.62–1.61 includes both appreciable benefit and appreciable harm	None	HR 1.00 (0.62–1.61), p=0.99. 3-year locoregional recurrence 20.2% vs 17.4% → 28 more per 1,000, with an interval compatible with fewer or more. Equivalence should not be inferred	⊕⊕◯◯ LOW (high, −1 indirectness, −1 imprecision)
Grade ≥3 adverse events (CRITICAL — 8)	1 direct RCT (ESOPEC); 438	Not serious	Not assessable — single trial	Serious (−1) — adverse-event definitions and exposure windows differ between a perioperative chemotherapy regimen and a preoperative chemoradiotherapy regimen, precluding a like-for-like comparison	Cannot be assessed — no pooled or domain-level estimate with confidence intervals is reported; the comparison is narrative	None	Distinct toxicity profiles rather than a single comparative estimate: higher haematological and neurological toxicity preoperatively with FLOT (neutropenia, neuropathy, diarrhoea); higher radiation-related toxicity with CROSS	⊕⊕⊕◯ MODERATE (high, −1 indirectness)
90-day postoperative mortality (CRITICAL — 8)	1 direct RCT (ESOPEC); 438 (ITT)	Not serious — objective endpoint	Not assessable — single trial	Serious (−1) — 25 German high-volume centres; postoperative mortality is strongly dependent on centre volume and may not transfer to lower-volume settings	Serious (−1) — few events and the difference did not reach statistical significance	None	3.1% vs 5.6% → 25 fewer deaths per 1,000; difference not statistically significant	⊕⊕◯◯ LOW (high, −1 indirectness, −1 imprecision)
R0 resection rate (IMPORTANT — 6)	1 direct RCT (ESOPEC); 438	Not serious	Not assessable — single trial	Serious (−1) — 25 German high-volume centres	Cannot be assessed until the estimate is extracted	None	[Rates, difference and 95% CI to be extracted from the ESOPEC primary publication]	Not rated pending extraction of the effect estimate
Health-related quality of life/patient-reported outcomes (CRITICAL — 8)	1 conference report of ESOPEC (2026); number of respondents not stated in the abstract	Not serious as designed (pre-specified PRO analysis within a randomised trial), but cannot be fully appraised from an abstract	Not assessable — single contributing report	Serious (−1) — instruments (QLQ-C30, QLQ-OES18, CIPN20) reported without domain-level tables or completion rates	Very serious (−2) — no effect estimates or confidence intervals are available, so precision cannot be established	Available only as a conference abstract, without peer review or longitudinal analysis	Not estimable from the available report	⊕◯◯◯ VERY LOW (high, −1 indirectness, −2 imprecision)

GRADE certainty: ⊕⊕⊕⊕ high, ⊕⊕⊕◯ moderate, ⊕⊕◯◯ low, ⊕◯◯◯ very low. Importance is rated on the GRADE 1–9 scale (7–9 critical, 4–6 important). All outcomes derive from randomised evidence and therefore start at high certainty; the rating shown equals high minus the downgrades listed in that row, each of which is stated explicitly. Inconsistency is recorded as “not assessable” wherever a single study contributes the estimate, because between-study heterogeneity cannot be evaluated from one study; single-trial evidence is instead reflected under indirectness and, where the confidence interval permits clinically divergent conclusions, under imprecision. Absolute effects are derived from the reported event rates in the ESOPEC intention-to-treat or resected population as indicated. Neo-AEGIS was not pooled with ESOPEC because its comparator arm combined ECF, ECX and FLOT. ITT, intention-to-treat; HR, hazard ratio; pCR, pathological complete response; PRO, patient-reported outcome.

The certainty of evidence for the principal clinical outcomes was assessed using the GRADE (Grading of Recommendations Assessment, Development and Evaluation) framework. Certainty ratings considered risk of bias, inconsistency, indirectness, imprecision, and publication bias where applicable. Given that direct comparative evidence between perioperative FLOT and neoadjuvant CROSS derives largely from a single phase III randomised trial (ESOPEC), inconsistency could not be evaluated for those outcomes informed by ESOPEC alone: with a single contributing study there is no between-study heterogeneity to assess, and the previous designation of “serious inconsistency” for these outcomes has been corrected to “not assessable”. Where evidence rested on a single trial, on a population restricted to resected patients rather than the intention-to-treat population, or on outcome definitions that differed between contributing reports, certainty was instead downgraded once for indirectness. Locoregional recurrence was additionally downgraded for imprecision, because the confidence interval around the hazard ratio is compatible with a clinically relevant difference in either direction. Each rating in **Table 7** therefore begins at high certainty (randomised evidence) and reflects the number of downgrades explicitly listed for that outcome. The resulting certainty assessments are presented in **Table 7** and **SupplementaryAppendix S2**.

### 4.2 Contextualising Neo-AEGIS and ESOPEC: a synthesis

The absence of survival differences in Neo-AEGIS and the significant OS benefit in ESOPEC appear contradictory but are reconcilable. The regimen heterogeneity hypothesis (dilution by ECF/ECX) is framed as a plausible but unverified explanation. Neo-AEGIS does not report FLOT-specific survival estimates or treatment-by-regimen interaction testing; therefore, it cannot be established from Neo-AEGIS data that the neutral result was caused by ECF/ECX dilution. Alternative explanations include: the protocol amendment from superiority to non-inferiority (changing the statistical framework mid-trial); the smaller sample size; differences in surgical volume and centre expertise; and treatment-era effects. ESOPEC’s design — pre-specifying FLOT as the intervention in all patients — avoids this ambiguity and provides a cleaner comparison.

### 4.3 The CROSS plus nivolumab pathway

The contemporary relevance of the CROSS regimen should be interpreted in light of the findings of the CheckMate 577 trial, which demonstrated a significant improvement in disease-free survival with adjuvant nivolumab following neoadjuvant chemoradiotherapy and R0 resection in patients without a pathological complete response (22.4 vs. 11.0 months; HR 0.69). Consequently, CROSS followed by surgery and adjuvant nivolumab represents an evidence-based multimodal treatment pathway. Importantly, adjuvant nivolumab was not incorporated into the CROSS arm of the ESOPEC trial. Therefore, the comparison performed in ESOPEC reflects perioperative FLOT versus CROSS alone rather than the contemporary CROSS-plus-nivolumab strategy. Whether the overall survival advantage observed with FLOT would be maintained when compared with this modern treatment sequence remains unknown and warrants prospective evaluation. This limitation should be considered when interpreting the applicability of ESOPEC findings to current clinical practice.

### 4.4 External validity and clinical applicability

ESOPEC was conducted exclusively in 25 German centres with established high-volume oesophagectomy programmes, standardised surgical techniques, and experienced multidisciplinary teams. The external validity of these results to healthcare systems with lower surgical volume, less experienced teams, or less standardised oncological care is uncertain. The real-world CROSS data (median OS 33.7 months vs. 37 months in ESOPEC) illustrate the magnitude of the efficacy-effectiveness gap even for an established protocol.

Multiple clinically legitimate scenarios may favour CROSS: (1) patients with high-risk surgical anatomy or proximal tumour location favoring locoregional sterilisation; (2) patients with significant dysphagia requiring rapid relief, where the radiosensitising effect of concurrent radiation may be particularly advantageous; (3) limited access to FLOT administration infrastructure; (4) patient preference after informed discussion of toxicity profiles; and (5) patients enrolled in trials combining CROSS with adjuvant nivolumab. Treatment selection must be individualised within multidisciplinary teams, incorporating tumour characteristics, patient fitness, institutional expertise, and patient values.

### 4.5 Biomarker integration and future directions

None of the included trials prospectively evaluated predictive biomarkers (HER2, PD-L1, microsatellite instability, circulating tumour DNA) as determinants of differential response to FLOT versus CROSS. This represents a major unmet need. Future trials should incorporate mandatory biomarker stratification. Ongoing phase III trials (KEYNOTE-585, MATTERHORN, DANTE) are evaluating FLOT in combination with immune checkpoint inhibitors. If positive, these trials may further compound the systemic control advantage of FLOT-based regimens. Adaptive strategies — such as ctDNA-guided switching between FLOT and CROSS based on early biological response — represent a conceptually attractive but currently investigational approach.

Unlike previous reviews, the present review distinguishes direct, indirect, observational and contextual evidence, incorporates the ESOPEC recurrence analysis, evaluates certainty of evidence using GRADE, and discusses applicability of the contemporary CROSS–nivolumab pathway.

### 4.6 Limitations

The principal limitations of this review are: (1) the evidence base for the direct FLOT vs. CROSS comparison rests primarily on a single phase III RCT (ESOPEC); (2) the narrative synthesis precludes quantitative pooling of effect estimates; (3) the review was not prospectively registered; (4) ESOPEC enrolled exclusively patients treated in high-volume German centres, limiting generalisability; (5) the postoperative FLOT component was not completed in approximately 26% of resected patients, raising practical concerns about real-world delivery; (6) patient-reported and quality-of-life outcomes for the direct comparison are available only in conference-abstract form and remain incompletely reported; (7) the contemporary CROSS-plus-nivolumab pathway is not represented in any direct comparison with FLOT; and (8) Scopus and Web of Science were searched for the original submission but were removed *post hoc* during revision, so the final search rests on three bibliographic databases and the possibility of a small number of uniquely indexed records being missed cannot be excluded.

## 5 Conclusion

ESOPEC provides the strongest currently available evidence supporting perioperative FLOT over neoadjuvant CROSS in eligible patients with resectable lower oesophageal adenocarcinoma, with improved OS (HR 0.70; p=0.012) and a likely reduction in distant recurrence (HR 0.59; p<0.001), while no difference in locoregional control was detected (HR 1.00, 95% CI 0.62–1.61), a result whose confidence interval does not permit a conclusion of equivalence. GRADE certainty for the OS benefit is rated moderate: FLOT is likely, but not definitively established as, superior to CROSS, given the single open-label trial design and geographic restriction to high-volume German centres. CROSS retains clinical validity for patients with performance limitations, local anatomical considerations, limited access to FLOT infrastructure, or as a foundation for adjuvant nivolumab in non-complete responders. Whether FLOT maintains its OS advantage over the contemporary CROSS-plus-nivolumab sequence is an open and clinically critical question that requires dedicated prospective evaluation. Treatment selection must be individualised within a multidisciplinary oncological framework, guided by tumour stage, histological and molecular features, patient fitness, institutional expertise, and patient preference.

## Data Availability

The original contributions presented in the study are included in the article/**Supplementary Material**. Further inquiries can be directed to the corresponding author.
