# Supplementary material for: Perioperative FLOT chemotherapy versus neoadjuvant chemoradiotherapy (CROSS protocol) in locally advanced adenocarcinoma of the lower oesophagus and oesophagogastric junction (Siewert types I–II): systematic review and narrative synthesis (SWiM)
Source: Front Oncol. 2026 Sep 15;16:1887422. doi: 10.3389/fonc.2026.1887422 (PMC13619382; doi:10.3389/fonc.2026.1887422)
Supplement: Supplementary file 1 [file Table1.docx]

**Supplementary Appendix S1**

**Search Strategies, Deduplication Log, Screening Summary, and Full-Text Exclusions**

# **S1.1 Database-Specific Search Strategies**

Three databases were searched from January 2012 through 30 June 2026 (last run 30 June 2026). Scopus and Web of Science were not searched. Each strategy combined an oesophageal/oesophagogastric-junction adenocarcinoma block, an intervention block (FLOT and CROSS/chemoradiotherapy), and a timing block, with study-design and date/language limits.

## **PubMed / MEDLINE (n = 1,387)**

```
(
  "Esophageal Neoplasms"[MeSH] OR "Adenocarcinoma"[MeSH] OR
  "esophageal adenocarcinoma"[TIAB] OR "oesophageal adenocarcinoma"[TIAB] OR
  "gastroesophageal junction adenocarcinoma"[TIAB] OR
  "gastro-oesophageal junction"[TIAB] OR "Siewert"[TIAB] OR
  "lower esophagus"[TIAB] OR "lower oesophagus"[TIAB] OR
  "Barrett adenocarcinoma"[TIAB] OR "esophagogastric junction"[TIAB]
)
AND
(
  "FLOT"[TIAB] OR "fluorouracil leucovorin oxaliplatin docetaxel"[TIAB] OR
  "perioperative chemotherapy"[TIAB] OR
  "Antineoplastic Combined Chemotherapy Protocols"[MeSH] OR
  "CROSS"[TIAB] OR "chemoradiotherapy"[TIAB] OR "chemoradiation"[TIAB] OR
  "carboplatin paclitaxel radiotherapy"[TIAB] OR
  "neoadjuvant chemoradiotherapy"[TIAB] OR "Chemoradiotherapy"[MeSH]
)
AND
( "neoadjuvant"[TIAB] OR "perioperative"[TIAB] OR "preoperative"[TIAB] OR
  "Neoadjuvant Therapy"[MeSH] )
AND
( "randomized controlled trial"[pt] OR "comparative study"[pt] OR
  "cohort study"[TIAB] OR "meta-analysis"[pt] OR "systematic review"[TIAB] )
AND ("2012/01/01"[PDAT] : "2026/06/30"[PDAT]) AND English[lang]
```

**Filters: editorials, letters, and case reports excluded. Records identified: n = 1,387.**

## **Embase (n = 968)**

```
('esophageal cancer'/exp OR 'esophagus adenocarcinoma'/exp OR
 'gastroesophageal junction tumor'/exp OR 'barrett esophagus'/exp OR
 'esophageal adenocarcinoma':ab,ti OR 'oesophageal adenocarcinoma':ab,ti OR
 'lower esophagus':ab,ti OR 'Siewert':ab,ti OR
 'gastroesophageal junction':ab,ti)
AND
('FLOT':ab,ti OR 'perioperative chemotherapy':ab,ti OR 'CROSS protocol':ab,ti OR
 'chemoradiotherapy'/exp OR 'neoadjuvant chemoradiotherapy':ab,ti OR
 ('carboplatin'/exp AND 'paclitaxel'/exp AND 'radiotherapy'/exp) OR
 'chemoradiation':ab,ti)
AND
('neoadjuvant therapy'/exp OR 'perioperative care'/exp OR 'neoadjuvant':ab,ti OR
 'perioperative':ab,ti OR 'preoperative treatment':ab,ti)
AND
('randomized controlled trial'/exp OR 'cohort analysis'/exp OR
 'comparative study'/exp OR 'meta analysis'/exp OR 'systematic review'/exp)
AND [2012-2026]/py AND [english]/lim
NOT ('letter'/it OR 'note'/it OR 'editorial'/it)
```

**Records identified: n = 968.**

## **Cochrane CENTRAL (n = 269)**

```
("esophageal adenocarcinoma" OR "oesophageal adenocarcinoma" OR
 "gastroesophageal junction adenocarcinoma" OR "gastro-oesophageal junction" OR
 "lower esophagus" OR "lower oesophagus" OR "Siewert" OR "Barrett adenocarcinoma")
AND
("FLOT" OR "fluorouracil leucovorin oxaliplatin docetaxel" OR
 "perioperative chemotherapy" OR "CROSS" OR "chemoradiotherapy" OR
 "neoadjuvant chemoradiotherapy" OR "carboplatin paclitaxel" OR "chemoradiation")
AND ("neoadjuvant" OR "perioperative" OR "preoperative")
```

**Records identified: n = 269.**

## **ClinicalTrials.gov**

Searched to identify ongoing/unpublished trials and to verify registration and protocol details (NCT02509286 ESOPEC; NCT01246960 Neo-AEGIS; NCT01522768 FLOT4; NCT01756040 CROSS follow-up; NCT04095182 KEYNOTE-585; NCT03416062 MATTERHORN; NCT02359968 DANTE). No additional eligible comparative studies were identified beyond those retrieved from the bibliographic databases.

| **Database** | **Date run** | **Records (n)** |
| --- | --- | --- |
| PubMed/MEDLINE | 30 Jun 2026 | 1,387 |
| Embase | 30 Jun 2026 | 968 |
| Cochrane CENTRAL | 30 Jun 2026 | 269 |
| Total | — | 2,624 |

# **S1.2 Deduplication Log**

The 2,624 records were imported into reference-management software. Duplicates were removed by matching on PubMed ID, then DOI, then normalised title. In total 311 duplicate records were removed (177 by PubMed ID, 103 by DOI, 31 by title), leaving 2,313 unique records for screening.

| **Matching criterion** | **Records removed** |
| --- | --- |
| PubMed ID match | 177 |
| DOI match (no/differing PMID) | 103 |
| Normalised title match | 31 |
| Total duplicates removed | 311 |
| Unique records retained (2,624 − 310) | 2,313 |

# **S1.3 Screening Summary**

The 2,314 unique records were screened by title and abstract, then at full text against the eligibility criteria. The cascade below reconciles with the PRISMA flow diagram (Figure 1).

| **Stage** | **Records in** | **Excluded** | **Records out** |
| --- | --- | --- | --- |
| Title/abstract screening | 2,313 | 2,266 | 48 |
| Full-text eligibility assessment | 48 | 35 | 13 |
| Distinct reports included (ESOPEC conference abstract counted once) | 13 | 1 | 11 |
| Underlying trials/cohorts | 13 reports | — | 9 trials and cohort |

*The 13 reports correspond to 8 underlying trials and cohorts (CROSS [van Hagen; Eyck]; FLOT4; Neo-AEGIS; ESOPEC [NEJM; ASCO abstract; JCO recurrence]; Gebauer; Defize; van Laarhoven; CheckMate 577), as detailed in Table 2 of the manuscript.*

# **S1.4 Full-Text Records Excluded, with Reasons (n = 36)**

## **Mixed histology without an extractable adenocarcinoma-specific subgroup (n = 14)**

| **#** | **First author (year)** | **Title** |
| --- | --- | --- |
| 1 | Garcia-Aguilar J 2022 | Organ Preservation in Patients With Rectal Adenocarcinoma Treated With Total Neoadjuvant T… |
| 2 | Janjigian YY 2025 | Perioperative Durvalumab in Gastric and Gastroesophageal Junction Cancer |
| 3 | Conroy T 2024 | Total neoadjuvant therapy with mFOLFIRINOX versus preoperative chemoradiotherapy in patien… |
| 4 | Bahadoer RR 2021 | Short-course radiotherapy followed by chemotherapy before total mesorectal excision (TME) … |
| 5 | Leong T 2024 | Preoperative Chemoradiotherapy for Resectable Gastric Cancer |
| 6 | Shitara K 2024 | Neoadjuvant and adjuvant pembrolizumab plus chemotherapy in locally advanced gastric or ga… |
| 7 | van der Wilk BJ 2025 | Neoadjuvant chemoradiotherapy followed by active surveillance versus standard surgery for … |
| 8 | Lorenzen S 2024 | Perioperative Atezolizumab Plus Fluorouracil, Leucovorin, Oxaliplatin, and Docetaxel for R… |
| 9 | Guo X 2025 | Neoadjuvant Chemoradiotherapy vs Chemoimmunotherapy for Esophageal Squamous Cell Carcinoma |
| 10 | Shapiro J 2015 | Neoadjuvant chemoradiotherapy plus surgery versus surgery alone for oesophageal or junctio… |
| 11 | Kato K 2024 | Doublet chemotherapy, triplet chemotherapy, or doublet chemotherapy combined with radiothe… |
| 12 | Versteijne E 2020 | Preoperative Chemoradiotherapy Versus Immediate Surgery for Resectable and Borderline Rese… |
| 13 | Hoeppner J 2016 | ESOPEC: prospective randomized controlled multicenter phase III trial comparing perioperat… |
| 14 | Shitara K 2025 | Pembrolizumab Plus Chemotherapy Versus Chemotherapy as Perioperative Therapy in Locally Ad… |

## **Single-arm design without a comparator (n = 11)**

| **#** | **First author (year)** | **Title** |
| --- | --- | --- |
| 15 | Janssen QP 2025 | Neoadjuvant FOLFIRINOX versus neoadjuvant gemcitabine-based chemoradiotherapy in resectabl… |
| 16 | Lin ZY 2024 | Neoadjuvant short-course radiotherapy followed by camrelizumab and chemotherapy in locally… |
| 17 | Huang TC 2015 | Systematic review and network meta-analysis: neoadjuvant chemoradiotherapy for locoregiona… |
| 18 | Noordman BJ 2018 | Detection of residual disease after neoadjuvant chemoradiotherapy for oesophageal cancer (… |
| 19 | Fokas E 2019 | Randomized Phase II Trial of Chemoradiotherapy Plus Induction or Consolidation Chemotherap… |
| 20 | Yang H 2018 | Neoadjuvant Chemoradiotherapy Followed by Surgery Versus Surgery Alone for Locally Advance… |
| 21 | Leong T 2017 | TOPGEAR: A Randomized, Phase III Trial of Perioperative ECF Chemotherapy with or Without P… |
| 22 | Yang H 2021 | Long-term Efficacy of Neoadjuvant Chemoradiotherapy Plus Surgery for the Treatment of Loca… |
| 23 | Al-Batran SE 2016 | Histopathological regression after neoadjuvant docetaxel, oxaliplatin, fluorouracil, and l… |
| 24 | SPACE-FLOT Investigators. 2025 | Pathological response guides adjuvant 5-fluorouracil, leucovorin, oxaliplatin, and docetax… |
| 25 | Ghaneh P 2023 | Immediate surgery compared with short-course neoadjuvant gemcitabine plus capecitabine, FO… |

## **Non-resectable or metastatic disease population (n = 10)**

| **#** | **First author (year)** | **Title** |
| --- | --- | --- |
| 26 | Liu Y 2023 | Efficacy and safety of neoadjuvant immunotherapy combined with chemoradiotherapy or chemot… |
| 27 | Lordick F 2025 | Adjuvant immunotherapy in patients with resected gastric and oesophagogastric junction can… |
| 28 | Liu L 2025 | Comparative study of neoadjuvant chemoradiotherapy, chemotherapy, and chemoimmunotherapy f… |
| 29 | Cercek A 2018 | Adoption of Total Neoadjuvant Therapy for Locally Advanced Rectal Cancer |
| 30 | Ronellenfitsch U 2024 | Preoperative Chemoradiotherapy vs Chemotherapy for Adenocarcinoma of the Esophagogastric J… |
| 31 | Baltatzis M 2020 | Neoadjuvant chemoradiotherapy before resection of perihilar cholangiocarcinoma: A systemat… |
| 32 | Yang Y 2025 | Neoadjuvant Immuno-Chemotherapy Versus Neoadjuvant Chemoradiotherapy in Locally Advanced O… |
| 33 | Yu YK 2024 | Neoadjuvant chemotherapy combined with immunotherapy versus neoadjuvant chemoradiotherapy … |
| 34 | Tang H 2023 | Neoadjuvant chemoradiotherapy versus neoadjuvant chemotherapy followed by minimally invasi… |
| 35 | Zhu Y 2017 | Meta-Analysis for the Therapeutic Effect of Neoadjuvant Therapy in Resectable Esophageal C… |

## **Insufficient outcome reporting (n = 1)**

| **#** | **First author (year)** | **Title** |
| --- | --- | --- |
| 36 | Lin JP 2025 | Neoadjuvant chemoimmunotherapy versus neoadjuvant chemoradiotherapy in patients with borde… |

*Records were identified predominantly through PubMed/MEDLINE; database of origin was recorded for each. A record-level export (identifier, decision, and reason) is available from the corresponding author on reasonable request.*
