# Supplementary material for: Perioperative FLOT chemotherapy versus neoadjuvant chemoradiotherapy (CROSS protocol) in locally advanced adenocarcinoma of the lower oesophagus and oesophagogastric junction (Siewert types I–II): systematic review and narrative synthesis (SWiM)
Source: Front Oncol. 2026 Sep 15;16:1887422. doi: 10.3389/fonc.2026.1887422 (PMC13619382; doi:10.3389/fonc.2026.1887422)
Supplement: Supplementary file 2 [file Table2.docx]

**Supplementary Appendix S2**

**Risk-of-Bias Worksheets (RoB 2 / ROBINS-I) and GRADE Evidence Profile**

This appendix contains: **(S2.1)** completed-form worksheets for RoB 2 (randomised trials) and ROBINS-I (non-randomised studies), presenting the signalling questions domain by domain; and **(S2.2)** a GRADE evidence profile in which every downgrading decision is stated explicitly with its rationale.

**S2.1 Risk-of-Bias Worksheets**

**A. RoB 2 — Signalling questions (randomised trials)**

**ESOPEC (Hoeppner 2025) — overall: LOW**

| **Domain** | **Signalling question** | **Answer** | **Domain judgement** | **Rationale** |
| --- | --- | --- | --- | --- |
| D1. Randomisation process | 1.1 Was the allocation sequence random? | Y | Low | Central web-based block randomisation stratified by center, T-stage, and N-stage ensured a truly random sequence. |
|  | 1.2 Was the allocation sequence concealed until participants were enrolled and assigned? | Y |  | Allocation was centrally concealed via an automated system prior to assignment. |
|  | 1.3 Did baseline differences between groups suggest a problem with the randomisation process? | N |  | Baseline clinical characteristics, age, and tumor staging were well balanced between arms. |
| D2. Deviations from intended interventions | 2.1 Were participants aware of their assigned intervention during the trial? | Y | Low | Due to the nature of surgery and chemoradiotherapy vs chemotherapy, blinding was not feasible; open-label design was used. |
|  | 2.2 Were carers and people delivering the interventions aware of assignment? | Y |  | Carers and surgical teams were unblinded. |
|  | 2.3 Were there deviations from the intended intervention that arose because of the trial context? | N |  | Deviations were minimal and typical of oncology trials without preferential contextual impact on either arm. |
|  | 2.4 Was an appropriate analysis used to estimate the effect of assignment to intervention (ITT)? | Y |  | Primary overall survival (OS) and progression-free survival (PFS) analyses were performed on the full intention-to-treat (ITT) population (n=438). |
| D3. Missing outcome data | 3.1 Were data for this outcome available for all, or nearly all, participants randomised? | Y | Low | Survival tracking in the ITT cohort was nearly 100% complete at median follow-up of 55 months. |
|  | 3.2 Is there evidence that the result was not biased by missing outcome data? | Y |  | Minimal dropouts/withdrawals occurred across both intervention groups. |
|  | 3.3 Could missingness in the outcome depend on its true value? | N |  | Missingness was negligible and unrelated to true mortality status. |
| D4. Measurement of the outcome | 4.1 Was the method of measuring the outcome inappropriate? | N | Low | Overall survival (all-cause mortality) is an objective, unequivocal endpoint. |
|  | 4.2 Could measurement of the outcome have differed between groups? | N |  | Survival status ascertainment was standardized across both trial arms. |
|  | 4.3 Were outcome assessors aware of the intervention received? | Y |  | Assessors were unblinded, but death dates are immune to observer subjectivity. |
|  | 4.4 Could assessment of the outcome have been influenced by knowledge of intervention received? | N |  | Knowledge of treatment assignment cannot alter the measurement of overall mortality. |
| D5. Selection of the reported result | 5.1 Were the data analysed in accordance with a pre-specified plan finalised before unblinded outcome data were available? | Y | Low | Trial was prospectively registered (NCT02509286), and primary endpoints matched the protocol. |
|  | 5.2 Is the numerical result likely to have been selected from multiple eligible outcome measurements? | N |  | Single primary outcome measure (OS) was predefined and reported as planned. |
|  | 5.3 ... or from multiple eligible analyses of the data? | N |  | Unadjusted ITT Cox proportional-hazards models were executed per protocol. |

**Neo-AEGIS (Reynolds 2023) — overall: SOME CONCERNS**

| **Domain** | **Signalling question** | **Answer** | **Domain judgement** | **Rationale** |
| --- | --- | --- | --- | --- |
| D1. Randomisation process | 1.1 Was the allocation sequence random? | Y | Low | Randomisation was centrally managed using a computer-generated sequence. |
|  | 1.2 Was the allocation sequence concealed until participants were enrolled and assigned? | Y |  | Allocation was concealed prior to enrollment via central registration. |
|  | 1.3 Did baseline differences between groups suggest a problem with the randomisation process? | N |  | Baseline parameters were comparable between the CROSS and perioperative chemotherapy arms. |
| D2. Deviations from intended interventions | 2.1 Were participants aware of their assigned intervention during the trial? | Y | Some concerns | Open-label design was used. The chemotherapy arm backbone was modified during the trial (transition from modified MAGIC to FLOT), introducing protocol-driven delivery heterogeneity. |
|  | 2.2 Were carers and people delivering the interventions aware of assignment? | Y |  | Clinical personnel were unblinded. |
|  | 2.3 Were there deviations from the intended intervention that arose because of the trial context? | PY |  | Regimen changes mid-study altered treatment intensity for a subset of participants. |
|  | 2.4 Was an appropriate analysis used to estimate the effect of assignment to intervention (ITT)? | Y |  | Analysis was conducted on the intention-to-treat population. |
| D3. Missing outcome data | 3.1 Were data for this outcome available for all, or nearly all, participants randomised? | Y | Low | Follow-up for the primary survival endpoint was comprehensive. |
|  | 3.2 Is there evidence that the result was not biased by missing outcome data? | Y |  | Loss to follow-up was minimal and balanced. |
|  | 3.3 Could missingness in the outcome depend on its true value? | N |  | Missing outcome data were negligible. |
| D4. Measurement of the outcome | 4.1 Was the method of measuring the outcome inappropriate? | N | Low | Overall survival is an objective, standardized endpoint. |
|  | 4.2 Could measurement of the outcome have differed between groups? | N |  | Survival status determination was identical in both arms. |
|  | 4.3 Were outcome assessors aware of the intervention received? | Y |  | Assessors were unblinded. |
|  | 4.4 Could assessment of the outcome have been influenced by knowledge of intervention received? | N |  | Mortality assessment is objective and not subject to assessor bias. |
| D5. Selection of the reported result | 5.1 Were the data analysed in accordance with a pre-specified plan finalised before unblinded outcome data were available? | PN | Some concerns | The trial protocol was formally amended mid-study from a superiority design to a non-inferiority design, altering the statistical analysis framework. |
|  | 5.2 Is the numerical result likely to have been selected from multiple eligible outcome measurements? | N |  | OS was reported as primary. |
|  | 5.3 ... or from multiple eligible analyses of the data? | PY |  | The trial design shift complicates post-hoc superiority vs non-inferiority interpretations. |

**FLOT4 (Al-Batran 2019) — overall: LOW**

| **Domain** | **Signalling question** | **Answer** | **Domain judgement** | **Rationale** |
| --- | --- | --- | --- | --- |
| D1. Randomisation process | 1.1 Was the allocation sequence random? | Y | Low | Central web-based 1:1 randomisation with dynamic classification. |
|  | 1.2 Was the allocation sequence concealed until participants were enrolled and assigned? | Y |  | Central allocation concealed assignment until registration. |
|  | 1.3 Did baseline differences between groups suggest a problem with the randomisation process? | N |  | Demographic and clinical factors were well balanced between FLOT and ECF/ECX arms. |
| D2. Deviations from intended interventions | 2.1 Were participants aware of their assigned intervention during the trial? | Y | Low | Open-label trial due to different intravenous/oral chemotherapy administration schedules. |
|  | 2.2 Were carers and people delivering the interventions aware of assignment? | Y |  | Treating clinicians were aware of assigned arm. |
|  | 2.3 Were there deviations from the intended intervention that arose because of the trial context? | N |  | Intervention protocols were delivered as intended without differential context deviations. |
|  | 2.4 Was an appropriate analysis used to estimate the effect of assignment to intervention (ITT)? | Y |  | Full ITT analysis was performed for all primary survival endpoints. |
| D3. Missing outcome data | 3.1 Were data for this outcome available for all, or nearly all, participants randomised? | Y | Low | Survival status was available for >98% of randomised patients. |
|  | 3.2 Is there evidence that the result was not biased by missing outcome data? | Y |  | Rates of missingness were low and balanced between arms. |
|  | 3.3 Could missingness in the outcome depend on its true value? | N |  | Outcome missingness was minimal. |
| D4. Measurement of the outcome | 4.1 Was the method of measuring the outcome inappropriate? | N | Low | Standardized robust assessment of overall survival. |
|  | 4.2 Could measurement of the outcome have differed between groups? | N |  | Follow-up schedules were equivalent across study arms. |
|  | 4.3 Were outcome assessors aware of the intervention received? | Y |  | Assessors were unblinded. |
|  | 4.4 Could assessment of the outcome have been influenced by knowledge of intervention received? | N |  | Objective endpoint (all-cause mortality) minimizes risk of measurement bias. |
| D5. Selection of the reported result | 5.1 Were the data analysed in accordance with a pre-specified plan finalised before unblinded outcome data were available? | Y | Low | Prospectively registered (NCT01522768) and analyzed according to the pre-specified statistical plan. |
|  | 5.2 Is the numerical result likely to have been selected from multiple eligible outcome measurements? | N |  | Standard predefined endpoints were reported. |
|  | 5.3 ... or from multiple eligible analyses of the data? | N |  | Analyses strictly followed the prespecified protocol. |

**CROSS (van Hagen 2012 / Eyck 2021) — overall: LOW**

| **Domain** | **Signalling question** | **Answer** | **Domain judgement** | **Rationale** |
| --- | --- | --- | --- | --- |
| D1. Randomisation process | 1.1 Was the allocation sequence random? | Y | Low | Computer-generated central randomisation with minimization based on center and staging. |
|  | 1.2 Was the allocation sequence concealed until participants were enrolled and assigned? | Y |  | Sequence assignment was centrally concealed until enrollment. |
|  | 1.3 Did baseline differences between groups suggest a problem with the randomisation process? | N |  | Baseline characteristics were evenly distributed between chemoradiotherapy + surgery vs surgery alone arms. |
| D2. Deviations from intended interventions | 2.1 Were participants aware of their assigned intervention during the trial? | Y | Low | Open-label trial comparing trimodality therapy against surgery alone. |
|  | 2.2 Were carers and people delivering the interventions aware of assignment? | Y |  | Treating clinicians were unblinded. |
|  | 2.3 Were there deviations from the intended intervention that arose because of the trial context? | N |  | Protocol adherence was exceptionally high without context-related deviations. |
|  | 2.4 Was an appropriate analysis used to estimate the effect of assignment to intervention (ITT)? | Y |  | Analysis performed on the ITT population for primary and long-term 10-year follow-up reports. |
| D3. Missing outcome data | 3.1 Were data for this outcome available for all, or nearly all, participants randomised? | Y | Low | Minimal loss to follow-up through 10-year extended monitoring. |
|  | 3.2 Is there evidence that the result was not biased by missing outcome data? | Y |  | Complete survival accounting across both trial arms. |
|  | 3.3 Could missingness in the outcome depend on its true value? | N |  | Outcome missingness was negligible. |
| D4. Measurement of the outcome | 4.1 Was the method of measuring the outcome inappropriate? | N | Low | Overall survival is an objective, standardized endpoint. |
|  | 4.2 Could measurement of the outcome have differed between groups? | N |  | Outcome measurement protocol was robust and identical in both arms. |
|  | 4.3 Were outcome assessors aware of the intervention received? | Y |  | Assessors were unblinded. |
|  | 4.4 Could assessment of the outcome have been influenced by knowledge of intervention received? | N |  | Measurement of death is objective and free from observer bias. |
| D5. Selection of the reported result | 5.1 Were the data analysed in accordance with a pre-specified plan finalised before unblinded outcome data were available? | Y | Low | Prespecified analysis plan registered (NTR1034 / ISRCTN48823278) and strictly followed. |
|  | 5.2 Is the numerical result likely to have been selected from multiple eligible outcome measurements? | N |  | Results were reported as prospectively defined. |
|  | 5.3 ... or from multiple eligible analyses of the data? | N |  | No evidence of selective analysis selection. |

**B. ROBINS-I — Signalling questions (non-randomised studies)**

**Gebauer 2023 — overall: SERIOUS**

| **Domain** | **Signalling question** | **Answer** | **Domain judgement** | **Rationale** |
| --- | --- | --- | --- | --- |
| D1. Confounding | 1.1 Is there potential for confounding of the effect of intervention in this study? | Y | Serious | High potential for confounding by indication, performance status, age, and baseline comorbidity in a non-randomized single-center cohort. |
|  | 1.2 Were participants analysed according to their starting intervention throughout follow-up? | PY |  | Patients remained in initial treatment groupings. |
|  | 1.3 Were the important confounding domains measured validly and reliably? | PN |  | Small sample size (n=76) restricted comprehensive measurement of confounding variables. |
|  | 1.4 Did the authors use an appropriate analysis method to control for confounding? | N |  | Unadjusted or minimally adjusted comparison without propensity score matching or multivariable regression. |
| D2. Selection of participants | 2.1 Was selection into the study related to intervention and outcome? | PY | Moderate | Single-center retrospective inclusion introduces substantial selection bias regarding treatment choice. |
|  | 2.2 Do start of follow-up and start of intervention coincide for most participants? | Y |  | Follow-up began at the initiation of neoadjuvant/perioperative therapy. |
|  | 2.3 Were adjustment techniques used to correct for selection biases? | N |  | No adjustment methods were applied to correct for institutional selection practices. |
| D3. Classification of interventions | 3.1 Were intervention groups clearly defined? | Y | Low | Regimens (FLOT vs CROSS) were clearly documented in medical records. |
|  | 3.2 Was the information used to define groups recorded at the start of intervention? | Y |  | Regimen administration was defined at baseline. |
|  | 3.3 Could classification of intervention status have been affected by knowledge of the outcome? | N |  | Interventions occurred prior to outcome measurement. |
| D4. Deviations from intended interventions | 4.1 Were there deviations from the intended intervention beyond usual practice? | PY | Moderate | Uncontrolled real-world dose reductions and cycle omissions occurred frequently in routine care. |
|  | 4.2 Were these deviations unbalanced between groups and likely to affect the outcome? | PY |  | FLOT completion rates differed from CROSS compliance in routine clinical delivery. |
| D5. Missing data | 5.1 Were outcome data available for all, or nearly all, participants? | PY | Moderate | Single-center chart reviews suffer from variable follow-up completeness. |
|  | 5.2 Were participants excluded due to missing data on intervention status or confounders? | NI |  | Details on missing data exclusions were insufficiently reported. |
|  | 5.3 Is there evidence that results were robust to the presence of missing data? | N |  | No sensitivity analyses were conducted for missing data. |
| D6. Measurement of outcomes | 6.1 Could the outcome measure have been influenced by knowledge of the intervention received? | PN | Moderate | Survival is objective, but response/morbidity assessments were unblinded and retrospective. |
|  | 6.2 Were outcome assessors aware of the intervention received? | Y |  | Assessors had full access to medical records. |
|  | 6.3 Were the methods of outcome assessment comparable across intervention groups? | Y |  | Outcome parameters were gathered via identical chart review methods. |
| D7. Selection of the reported result | 7.1 Is the reported effect estimate likely to be selected from multiple outcome measurements? | PY | Moderate | Retrospective non-registered observational study with risk of selective outcome reporting. |
|  | 7.2 ... from multiple analyses of the intervention-outcome relationship? | PY |  | Potential for selective post-hoc subgroup or survival cut-point reporting. |
|  | 7.3 ... from different subgroups? | NI |  | Subgroup selection transparency was limited. |

**Defize 2022 — overall: MODERATE**

| **Domain** | **Signalling question** | **Answer** | **Domain judgement** | **Rationale** |
| --- | --- | --- | --- | --- |
| D1. Confounding | 1.1 Is there potential for confounding of the effect of intervention in this study? | Y | Moderate | Confounding by indication exists, but propensity score matching (PSM) was utilized to adjust for major covariates. |
|  | 1.2 Were participants analysed according to their starting intervention throughout follow-up? | Y |  | Patients were analyzed based on initial treatment intention in the matched cohorts (n=222, 111/arm). |
|  | 1.3 Were the important confounding domains measured validly and reliably? | Y |  | Key prognostic variables (age, stage, performance status) were captured for matching. |
|  | 1.4 Did the authors use an appropriate analysis method to control for confounding? | Y |  | Propensity score matching (1:1) was effectively implemented. |
| D2. Selection of participants | 2.1 Was selection into the study related to intervention and outcome? | PY | Moderate | Institutional treatment preference over time may introduce subtle temporal selection biases. |
|  | 2.2 Do start of follow-up and start of intervention coincide for most participants? | Y |  | Baseline start of treatment coincided with follow-up entry. |
|  | 2.3 Were adjustment techniques used to correct for selection biases? | Y |  | PSM mitigated selection differences between treatment groups. |
| D3. Classification of interventions | 3.1 Were intervention groups clearly defined? | Y | Low | Treatment protocols (FLOT vs CROSS) were clearly classified. |
|  | 3.2 Was the information used to define groups recorded at the start of intervention? | Y |  | Documented at treatment initiation. |
|  | 3.3 Could classification of intervention status have been affected by knowledge of the outcome? | N |  | Group assignment was established prior to outcome collection. |
| D4. Deviations from intended interventions | 4.1 Were there deviations from the intended intervention beyond usual practice? | PY | Moderate | Standard real-world deviations (dose reductions, post-op FLOT omission) occurred. |
|  | 4.2 Were these deviations unbalanced between groups and likely to affect the outcome? | PY |  | Postoperative chemotherapy completion was lower than preoperative chemoradiotherapy adherence. |
| D5. Missing data | 5.1 Were outcome data available for all, or nearly all, participants? | Y | Low | Data completeness was high within the tertiary center registry. |
|  | 5.2 Were participants excluded due to missing data on intervention status or confounders? | PN |  | Exclusions for missing baseline matching variables were low. |
|  | 5.3 Is there evidence that results were robust to the presence of missing data? | Y |  | Matched sample had near-complete primary outcome data. |
| D6. Measurement of outcomes | 6.1 Could the outcome measure have been influenced by knowledge of the intervention received? | PN | Moderate | Survival endpoints are objective; secondary response measures carried unblinded assessment risk. |
|  | 6.2 Were outcome assessors aware of the intervention received? | Y |  | Unblinded retrospective evaluation. |
|  | 6.3 Were the methods of outcome assessment comparable across intervention groups? | Y |  | Standard institutional follow-up protocols were applied to both cohorts. |
| D7. Selection of the reported result | 7.1 Is the reported effect estimate likely to be selected from multiple outcome measurements? | N | Low | Outcomes were reported consistently with the pre-specified comparative protocol. |
|  | 7.2 ... from multiple analyses of the intervention-outcome relationship? | N |  | Primary matched Cox regression models were presented transparently. |
|  | 7.3 ... from different subgroups? | N |  | Main cohort findings were highlighted without selective subgroup emphasis. |

**van Laarhoven 2025 — overall: MODERATE**

| **Domain** | **Signalling question** | **Answer** | **Domain judgement** | **Rationale** |
| --- | --- | --- | --- | --- |
| D1. Confounding | 1.1 Is there potential for confounding of the effect of intervention in this study? | Y | Moderate | Single-arm real-world cohort evaluating CROSS (n=4,765); unmeasured real-world clinical confounders exist. |
|  | 1.2 Were participants analysed according to their starting intervention throughout follow-up? | Y |  | All patients starting CROSS in the nationwide registry were evaluated. |
|  | 1.3 Were the important confounding domains measured validly and reliably? | Y |  | Nationwide cancer registry (IKNL/DUCA) comprehensively captures clinical stage and baseline demographics. |
|  | 1.4 Did the authors use an appropriate analysis method to control for confounding? | NA |  | Single-arm contextual effectiveness study; internal comparator control was not applicable. |
| D2. Selection of participants | 2.1 Was selection into the study related to intervention and outcome? | N | Low | Unselected, nationwide population-based registry coverage minimizes selection bias. |
|  | 2.2 Do start of follow-up and start of intervention coincide for most participants? | Y |  | Entry coincides with diagnosis/treatment start in the national registry. |
|  | 2.3 Were adjustment techniques used to correct for selection biases? | NA |  | Population-wide registry inclusion. |
| D3. Classification of interventions | 3.1 Were intervention groups clearly defined? | Y | Low | Standard CROSS chemoradiotherapy protocol classification. |
|  | 3.2 Was the information used to define groups recorded at the start of intervention? | Y |  | Prospectively recorded in national quality registries. |
|  | 3.3 Could classification of intervention status have been affected by knowledge of the outcome? | N |  | Registered at routine clinical delivery. |
| D4. Deviations from intended interventions | 4.1 Were there deviations from the intended intervention beyond usual practice? | N | Low | Reflects routine real-world practice across all Dutch surgical and oncological centers. |
|  | 4.2 Were these deviations unbalanced between groups and likely to affect the outcome? | NA |  | Single-arm context. |
| D5. Missing data | 5.1 Were outcome data available for all, or nearly all, participants? | Y | Low | Mandatory national death registry linkage yields near-100% survival follow-up. |
|  | 5.2 Were participants excluded due to missing data on intervention status or confounders? | N |  | Exclusions were minimal. |
|  | 5.3 Is there evidence that results were robust to the presence of missing data? | Y |  | High data integrity due to national registry infrastructure. |
| D6. Measurement of outcomes | 6.1 Could the outcome measure have been influenced by knowledge of the intervention received? | N | Low | Overall survival linked to municipal personal records database. |
|  | 6.2 Were outcome assessors aware of the intervention received? | NA |  | Automated registry linkage. |
|  | 6.3 Were the methods of outcome assessment comparable across intervention groups? | Y |  | Standardized national vital statistics assessment. |
| D7. Selection of the reported result | 7.1 Is the reported effect estimate likely to be selected from multiple outcome measurements? | N | Low | Standard median OS and survival curves reported per registry protocol. |
|  | 7.2 ... from multiple analyses of the intervention-outcome relationship? | N |  | Pre-specified epidemiological analysis plan. |
|  | 7.3 ... from different subgroups? | N |  | Total population outcomes presented primary. |

**S2.2 GRADE Evidence Profile with Explicit Downgrading Rationale**

Randomised evidence starts at **HIGH** certainty. Each domain is rated "not serious" (no downgrade), "serious" (−1 level), or "very serious" (−2 levels). The certainty ratings below are those reported in Table 5/6 of the manuscript; this profile makes the reason for each downgrade explicit. No outcome was upgraded.

| **Outcome** | **Studies (n)** | **Starting certainty** | **Risk of bias** | **Inconsistency** | **Indirectness** | **Imprecision** | **Publication bias** | **Final certainty** |
| --- | --- | --- | --- | --- | --- | --- | --- | --- |
| Overall survival | 1 RCT (ESOPEC), n=438 | High | Not serious — RoB 2 low across all domains | Serious* — single direct trial; indirect Neo-AEGIS data conflict | Not serious — direct FLOT vs CROSS comparison in the target population | Not serious — 95% CI 0.53–0.92 excludes the null | Undetected | MODERATE |
| Progression-free survival | 1 RCT (ESOPEC), n=438 | High | Not serious | Serious* — single-trial evidence | Not serious | Not serious — 95% CI 0.51–0.85 | Undetected | MODERATE |
| Pathological complete response | 1 RCT + 1 observational study | High | Not serious for the RCT | Not serious within ESOPEC | Serious* — pCR definitions differ across studies (ypT0ypN0 vs TRG/Mandard) | Not serious | Undetected | MODERATE |
| Distant recurrence | 1 pre-specified sub-analysis (ESOPEC), n=371 resected | High | Not serious | Serious* — no comparator RCT data | Serious* — resected-patient sub-analysis, not the ITT population | Not serious — HR 0.59, p<0.001 | Undetected | MODERATE |
| Locoregional recurrence | 1 pre-specified sub-analysis (ESOPEC), n=371 | High | Not serious | Serious* — single-study evidence | Serious* — sub-analysis population | Not serious | Undetected | MODERATE |
| Grade ≥ 3 adverse events | 1 RCT + observational data | High | Not serious for the RCT | Not serious | Not serious | Not serious | Undetected | MODERATE |

****Note on apparent double-downgrading:*** *Distant and locoregional recurrence are each rated serious for both inconsistency and indirectness, which would ordinarily imply LOW certainty. The review team judged that these two ratings arise from the same underlying limitation — that both estimates derive from one pre-specified sub-analysis of a single trial — and therefore applied a single overall downgrade, yielding MODERATE certainty.*

**Summary of downgrading decisions**

| **Domain** | **Outcomes downgraded** | **Reason** |
| --- | --- | --- |
| Risk of bias | None | RoB 2 judged low for ESOPEC across all five domains. |
| Inconsistency | OS, PFS, distant recurrence, locoregional recurrence | The direct comparison rests on a single trial; Neo-AEGIS provides conflicting indirect evidence. |
| Indirectness | pCR, distant recurrence, locoregional recurrence | Differing pCR definitions across studies; recurrence estimates derive from a resected-patient sub-analysis rather than the ITT population. |
| Imprecision | None | Confidence intervals for the principal outcomes exclude the null. |
| Publication bias | None | Not formally assessable with fewer than 10 studies; no evidence of selective publication for the registered pivotal trials. |
| Upgrading | None | No large-effect, dose-response, or plausible-confounding criteria were applied. |
